# The association between exposure to secondhand smoke and psychological symptoms among Chinese children

**DOI:** 10.1186/s12889-019-7006-8

**Published:** 2019-07-10

**Authors:** Hui Wang, Fei Li, Yunting Zhang, Fan Jiang, Jun Zhang

**Affiliations:** 1 MOE-Shanghai Key Laboratory of Children’s Environmental Health, Xin Hua Hospital Affiliated to Shanghai Jiao Tong University School of Medicine, Shanghai, 200092 China; 2 Child Health Advocacy Institute, Shanghai Children’s Medical Center Affiliated to Shanghai Jiao Tong University School of Medicine, Shanghai, 200127 China; 3 Department of Developmental and Behavioral Pediatrics, Shanghai Children’s Medical Center Affiliated to Shanghai Jiao Tong University School of Medicine, Shanghai, 200127 China

**Keywords:** Secondhand smoke exposure, Psychological symptoms, Childhood, Population-based study

## Abstract

**Background:**

The effect of secondhand smoke (SHS) exposure on child psychological problems remained inconclusive in previous studies. The aim of this study is to explore the association between SHS exposure and psychological symptoms in children.

**Methods:**

This population-based cross-sectional survey used cluster random probability sampling and recruited children aged 6–13 years in 26 primary schools in Shanghai, China, in 2014. Duration of SHS exposure in children were categorized as none, < 1 h/day, 1–2 h/day, ≥3 h/day. Psychological symptoms were assessed by the parental version of the Strengths and Difficulties Questionnaire (SDQ). We used logistic regression to estimate the adjusted associations of SHS exposure with total SDQ and its specific domains. Multiple imputation was used for handling missing data.

**Results:**

A total of 17,571 children completed this survey, with a response rate of 99.7%. SDQ scores were available for 15,344 participants, of whom 20.9% were not exposed to SHS, 63.0% exposed for < 1 h/day, 10.4% for 1–2 h/day, and 5.7% for ≥3 h/day. Compared to children not exposed to SHS, SHS exposure was positively associated with total SDQ score: OR 1.42, 95% confidence interval (CI) 1.22 to 1.66 for SHS exposure < 1 h/day, OR 2.14, 95% CI 1.76 to 2.62 for 1–2 h/day and OR 2.53, 95% CI 2.01 to 3.18 for ≥3 h/day after adjusting for sex, age, mode of birth, family socio-economic status and place of birth. These associations did not vary by sex.

**Conclusion:**

SHS exposure is an independent risk factor for psychological problems among children. Nonetheless, our study is unable to distinguish between fetal and child exposure to SHS.

**Electronic supplementary material:**

The online version of this article (10.1186/s12889-019-7006-8) contains supplementary material, which is available to authorized users.

## Background

Mental health disorders is the leading cause of health-related burden, representing for 15.5% of the disability adjusted life years in young people aged 10 to 24 years globally. [1] The prevalence of mental health disorders in children varies widely due to the heterogeneity in methodological and cultural factors; the estimated worldwide prevalence of mental health disorders is about 10 to 20% in children and is still increasing. [2] From a developmental perspective, childhood is being recognized as an important developmental transition stage during which many processes undergo substantial multi-dimensional changes including physiological, psychological and social. [3] Mental health disorders diagnosed in adulthood often originate in very early life. [4] Causal factors for mental health disorders are difficult to identify. Among the known or suspected risk factors, secondhand smoke (SHS) exposure is the major modifiable factor in the general population. [5] However, the mechanisms are still unclear. SHS exposure could be considered as an indicator for lower socio-economic status (SES), which in turn, is associated with poor mental health disorders. [6] The dopaminergic system could also be involved in the association between SHS exposure and mental health disorders and it is well established dopamine is an important neurotransmitter that controls many aspects of emotional, conduct and cognitive function. [7] Evidence from animal studies suggest that exposure to SHS increases dopamine release in brain areas such as caudate-putamen, nucleus accumbens and olfactory tubercle. [8] These brain areas are consistently demonstrated to be involved in emotional and behavioral regulation. [9]

Some previous studies have showed positive associations between SHS exposure and mental health disorders. [10–13] For example, one cross-sectional study in the US reported that a higher serum cotinine level increased the risk of depression, anxiety disorders, attention disorders and conduct problems after accounting for potential confounders among non-smoking children and adolescents. [10] However, another study has reported conflicting results in adults. [14]

Although smoke-free legislation has banned smoking in public areas in many parts of the world, this regulation is not applied to places such as homes or private cars. Smoking by parents or other household members are the primary source of child exposure to SHS. [15] In China, family structures are relatively complex, and the most common types are multigeneration and extended families. [16] About 40% of the world’s tobacco was consumed in China. [17] Therefore, Chinese children are more likely to be exposed to SHS at home. As such, evidence from the Chinese context could be valuable. We examined this issue in a large population-based cross-sectional survey, ‘the Shanghai Child Health, Education and lifestyle Evaluation (SCHEDULE) study’. We also examined whether these associations varied by sex.

## Methods

### Data source

The SCHEDULE study is a cross-sectional population-based survey in Shanghai, China, in June 2014. Detailed description is provided elsewhere. [18, 19] Briefly, seven districts were randomly selected from a total of 19 districts in Shanghai. Among these selected districts, 26 primary schools were randomly chosen. All students from grades one to five in the chosen schools were eligible for participation in this study. For schools with fewer than 1000 students, all of them were eligible, whereas in schools with over 1000 students, only half of the classes were randomly selected. All the students in the selected classes were eligible for this study. An invitation letter and a consent form were sent to the parents of the eligible students to inform them of the study and invite them to participate. If the parents or guardians agreed to join, they were asked to complete a self-administered questionnaire. Collected was information on parental demographic characteristics (parental education level and family income), perinatal characteristics of the index child (gestational age at birth, sex, mode of birth (vaginal delivery/caesarean delivery) and birth weight), SHS exposure status and offspring characteristics (medical history, food intake frequency, physical activity and mental health disorders during childhood). Anthropometric measures including weight and height were collected by trained researchers.

### Exposure

SHS exposure was reported by parents via the following questions: Is the child exposed to tobacco in private or public areas? and if yes: how long is the child exposed to SHS daily (< 1 h/day, 1–2 h/day and ≥ 3 h/day).

### Outcome

Psychological symptoms were assessed by the Strengths and Difficulties Questionnaires (SDQ) reported by parents in our study. [20] SDQ consists of a set of 25 items concerning the five most important domains of child behavioral and emotional problems including emotional symptoms, conduct problems, hyperactivity-inattention, peer problems and prosocial behavior. Each domain has 5 items, with each item scored 0 for not true, 1 for somewhat true, 2 for certainly true. The total difficulty score is the sum of the first four subscale scores (emotional symptoms, conduct problems, hyperactivity-inattention, peer problems), ranging from 0 to 40 with higher scores indicating more difficulties. The fifth subscale, i.e., prosocial behavior, describes positive attributes of the children. For this domain, the reverse coding was applied. SDQ has different language versions. The Chinese version of SDQ has the same structure as that in other languages and good reliability and validity. It has been widely used in China. [21] According to cut-off points recommended and widely used in China, [20–22] SDQ total and its subscales scores were classified into two groups as ‘abnormal’ and ‘normal’, and then subsequently categorized into “abnormal”, “borderline” and “normal” groups. Children classified into the “abnormal” group indicated that they have substantial risk of probable psychological symptoms. [21]

### Statistical analysis

Baseline characteristics by SHS exposure were compared using Pearson’s χ^2^ tests and analysis of covariance. Multivariable logistic regression was used to examine the adjusted associations of SHS exposure with total SDQ score and its specific domains in children. Whether the associations varied by sex was assessed based on the significance of interaction terms using likelihood ratio tests. A dose-response association was tested for linear trends across groups.

We select potential confounders that were associated with both exposure (i.e., SHS exposure) and outcomes (i.e., psychiatric symptoms). To illustrate the role of possible confounding, we present two models. Model 1 adjusted for sex and child age when the survey was conducted. Model 2 additionally adjusted for parental education, household income, mode of birth, place of birth and body mass index z-score (relative to the 2007 World Health Organization growth reference). [23]

Multiple imputation was used for missing exposures (SHS exposure was imputed for 5.4%) and confounders (paternal education imputed for 5.6%, maternal education for 6.4%, family income for 32.1%, mode of birth 9.3%, place of birth for 11.8%). Multiple imputation was specified based on a flexible additive regression with predictive mean matching incorporating information on all these factors including outcomes, exposures and other covariates (sex, child age when the survey was conducted, parental education, family income, place of birth, mode of birth and maternal gestational diabetes). [24] We summarized the results from 10 imputed datasets into single estimated odds ratio (OR) with confidence intervals (CI) adjusted for missing data uncertainty.

Finally, we conducted a sensitivity analysis using ordinal logistic regression model when psychological symptoms was categorized into three groups i.e. “normal”, “borderline” and “abnormal” to check for consistency. We also performed complete case analysis for comparison.

Data were analyzed using Stata version 13 (Stata Corp, College Station, Texas, USA) and R V.3.3.3 (R development Core Team, Vienna, Austria).

### Ethics approval

The SCHEDULLE study obtained ethical approval from the Institutional Review Board of the Shanghai Children’s Medical Center.

## Results

A total of 17,571 students participated in this population-based survey with the participation rate 99.7%. Of these 17,571, 15,344 participants had parent-reported SDQ scores measured at 6~13 ages and 10.9% had a psychological problem. Table 1 shows the total and the five domains of SDQ characteristics of these participants.

**Table 1:** Description of psychological symptoms among children in the SCHEDULE study in China

	Total SDQ score	Emotional symptoms	Conduct problems	Hyperactivity-attention	Peer-problems	Prosocial behavior
	N (%)	N (%)	N (%)	N (%)	N (%)	N (%)
Normal	11,678 (76.1)	12,928 (84.3)	12,258 (79.9)	10,775 (70.2)	7745 (50.5)	12,396 (81.6)
Borderline	1995 (13.0)	1217 (7.9)	1806 (11.8)	1616 (10.5)	3281 (21.4)	1680 (11.1)
Abnormal	1671 (10.9)	1199 (7.8)	1280 (8.3)	2953 (19.3)	4318 (28.1)	1155 (7.3)

Table 2 presents unadjusted associations between SHS exposure and potential confounders. Among the 15,344 participants, 20.9% children were not habitually exposed to SHS; 63.0% were exposed to SHS for < 1 h/day, 10.4% for 1–2 h/day, 5.7% for ≥3 h/day. Compared to children from low SES families, children from higher SES families are less prone to SHS exposure. SHS exposure was higher in children born in Shanghai than those born in other places across China. Higher SHS exposure level was associated with higher BMI.

**Table 2:** Baseline characteristics by secondhand smoke exposure from the SCHEDULE study in China

Secondhand smoke exposure/daily, %^a^						
Characteristics		None	< 1 h	1–2 h	≥3 h	*p* value
	N (%)	20.8	63.1	10.5	5.6	
Sex						0.968
Boys	9242 (53.8)	53.8	53.4	53.6	54.0	
Girls	7943 (46.2)	46.2	46.6	46.4	46.0	
Age (Mean score)		9.1	9.2	9.2	9.2	0.55
Household income						< 0.001
≤30,000	1564 (13.1)	9.1	13.4	16.1	13.3	
30,000-100,000	5306 (44.5)	35.2	46.6	48.4	44.7	
100,000-300,000	4190 (35.1)	42	34.3	30.6	35.2	
≥300,000	864 (7.3)	13.7	5.7	4.9	6.8	
Maternal education						< 0.001
Middle school or below	5594 (34.0)	24.9	36.3	37.1	29.3	
High school	4386 (26.7)	22.1	27.4	30.5	30.1	
College or above	6467 (39.3)	53.0	36.4	32.4	40.6	
Paternal education						< 0.001
Middle school or below	4834 (29.1)	19.4	31.1	34.5	25.8	
High school	5005 (30.2)	23.3	31.8	33.7	34.1	
College or above	6743 (40.7)	57.3	37.1	31.8	40.1	
Mode of birth						0.06
Vaginal	8012 (50.2)	48.8	50.3	52.5	48.4	
Cesarean	7933 (49.8)	51.2	49.7	47.5	51.6	
Place of birth						< 0.001
Shanghai	8096 (52.2)	48.8	52.9	55.0	57.4	
Others	7402 (47.8)	51.2	47.1	45.0	42.6	
BMI z-score (Mean score)		0.21	0.30	0.30	0.38	0.01

^a^ Given as % unless indicated

Table 3 shows the estimates of the contribution of SHS exposure to total SDQ score and its five specific domains. Compared to children without exposure, almost any duration of SHS exposure was associated with the total SDQ score: OR 1.42, 95% CI 1.22 to 1.66 for SHS exposure < 1 h/day, OR 2.14, 95% CI 1.76 to 2.62 for 1–2 h/day and OR 2.53, 95% CI 2.01 to 3.18 for ≥3 h/day after adjusting for sex and age. Likewise, any duration of SHS exposure were associated with five specific SDQ domains: emotional symptoms, conduct problems, hyperactivity-inattention, peer problems and prosocial behavior. After further adjustment for family SES, mode of birth, place of child birth and child BMI z-score, the associations for total SDQ score and other specific domains were attenuated slightly but remained significant. These associations did not vary by sex (*p* value for sex interaction 0.34 for general psychological problems, 0.97 for emotional symptoms, 0.68 for conduct problems, 0.79 for hyperactivity-inattention, 0.43 for peer relationship problems and 0.07 for prosocial behavior). The dose-response relationship was observed across the SHS exposure group (*p* value for linear trend < 0.001).

**Table 3:** Adjusted associations of secondhand smoke exposure with psychological symptoms using multiple imputation in the SCHEDULE study in China

	Secondhand smoke exposure			
	None	< 1 h/daily	1–2 h/daily	≥3 h/daily
	Reference	OR (95% CI)	OR (95% CI)	OR (95% CI)
Model 1				
Total SDQ score	1	1.60 (1.38 to 1.86)	2.44 (2.02 to 2.96)	2.75 (2.20 to 3.43)
Emotional symptoms	1	1.49 (1.26 to 1.76)	1.98 (1.59 to 2.48)	2.16 (1.66 to 2.81)
Conduct problems	1	1.46 (1.24 to 1.72)	2.03 (1.64 to 2.52)	2.20 (1.71 to 2.83)
Hyperactivity-inattention	1	1.37 (1.22 to 1.53)	1.79 (1.53 to 2.09)	2.14 (1.79 to 2.57)
Peer relationship problems	1	1.46 (1.33 to 1.61)	1.70 (1.48 to 1.94)	1.52 (1.29 to 1.80)
Prosocial behaviors	1	1.17 (0.99 to 1.38)	1.34 (1.06 to 1.68)	1.46 (1.11 to 1.91)
Model 2				
Total SDQ score	1	1.42 (1.22 to 1.66)	2.14 (1.76 to 2.62)	2.53 (2.01 to 3.18)
Emotional symptoms	1	1.38 (1.16 to 1.64)	1.84 (1.46 to 2.31)	2.05 (1.57 to 2.68)
Conduct problems	1	1.36 (1.15 to 1.61)	1.88 (1.50 to 2.34)	2.09 (1.61 to 2.70)
Hyperactivity-inattention	1	1.24 (1.10 to 1.39)	1.58 (1.35 to 1.86)	1.98 (1.65 to 2.38)
Peer relationship problems	1	1.25 (1.13 to 1.38)	1.41 (1.23 to 1.63)	1.36 (1.14 to 1.61)
Prosocial behaviors	1	1.06 (0.90 to 1.26)	1.14 (0.89 to 1.44)	1.37 (1.03 to 1.82)

Model 1 adjusted for sex, age at measurement;

Model 2 additionally adjusted for parents’ education, household income, mode of birth, place of birth and BMI z-score (relative to the 2007 World Health Organization growth reference)

When we used ordinal logistic regression, the results remained the same (Table 4). The proportional odds assumption for the SHS exposure was not violated with *p* values for likelihood ratio test ranging from 0.06 to 0.92. Complete case analysis showed a similar result (Additional file 1: Table S1).

**Table 4:** Adjusted associations of secondhand smoke exposure with psychological symptoms obtained from proportional odds model in the SCHEDULE study in China

	Secondhand smoke exposure			
	None	< 1 h/daily	1–2 h/daily	≥3 h/daily
	Reference	pOR (95% CI)	pOR (95% CI)	pOR (95% CI)
Model 1				
Total SDQ score	1	1.63 (1.47 to 1.81)	2.30 (2.00 to 2.65)	2.46 (2.08 to 2.92)
Emotional symptoms	1	1.41 (1.25 to 1.59)	1.94 (1.65 to 2.28)	1.75 (1.43 to 2.13)
Conduct problems	1	1.40 (1.26 to 1.57)	1.88 (1.63 to 2.19)	1.90 (1.59 to 2.28)
Hyperactivity-inattention	1	1.38 (1.26 to 1.52)	1.87 (1.64 to 2.13)	2.08 (1.77 to 2.44)
Peer relationship problems	1	1.45 (1.36 to 1.59)	1.73 (1.54 to 1.94)	1.53 (1.33 to 1.77)
Prosocial behaviors	1	1.28 (1.15 to 1.43)	1.48 (1.26 to 1.73)	1.31 (1.07 to 1.59)
Model 2				
Total SDQ score	1	1.47 (1.28 to 1.70)	1.98 (1.63 to 2.40)	2.28 (1.81 to 2.87)
Emotional symptoms	1	1.29 (1.10 to 1.53)	1.84 (1.48 to 2.28)	1.63 (1.24 to 2.14)
Conduct problems	1	1.40 (1.26 to 1.57)	1.88 (1.63 to 2.19)	1.90 (1.59 to 2.28)
Hyperactivity-inattention	1	1.20 (1.06 to 1.37)	1.52 (1.28 to 1.81)	1.99 (1.61 to 2.47)
Peer relationship problems	1	1.31 (1.17 to 1.46)	1.44 (1.23 to 1.69)	1.52 (1.25 to 1.85)
Prosocial behaviors	1	1.22 (1.05 to 1.42)	1.42 (1.15 to 1.76)	1.35 (1.03 to 1.77)

Model 1 adjusted for sex, age at measurement;

Model 2 additionally adjusted for parents’ education, household income, mode of birth, place of birth and BMI z-score (relative to the 2007 World Health Organization growth reference)

## Discussion

In this large population-representative study, we found that children who were exposed to any duration of SHS had a higher risk of having psychological symptoms and its specific domains concerning emotional symptoms, conduct problems, hyperactivity-inattention, peer relationship problems and prosocial behaviors than those who were not exposed to SHS after adjustment for potential confounders.

Our findings are consistent with most previous studies showing that children exposed to SHS had increased risks of general psychological symptoms and specific domains of psychological symptoms. [10, 11, 13, 25–27] Two studies have reported there existed a positive dose-response relationship between SHS exposure and psychological symptoms. [13, 27] Our results showing that SHS exposure associated with psychological symptoms concerning hyperactivity-inattention problems domain, are in line with another study using DSM-IV diagnosis criteria for attention deficit and hyperactivity disorder. [28] Our findings are partly consistent with two longitudinal studies which demonstrated that children exposed to pre- and postnatal tobacco were at remarkably increased risk of being classified as abnormal assessed by SDQ scale, although the SHS exposure during pregnancy seems to play a stronger role. [12, 29]

Despite that our study is a large population-based study with cluster random probability sampling, it has some limitations. First, SHS exposure was reported by parents, which was subject to inaccuracy and misclassification. However, a previous study conducted among school children confirmed that questionnaires reported by parents could provide a valid estimate of SHS exposure when comparing to serum cotinine levels. [30] Furthermore, underestimation of actual exposure could only make these estimates conservative. Meanwhile, we could not rule out the possibility that parents of children having psychological symptoms might have overreported SHS exposure which could overestimate the association. Second, as in other epidemiological studies, psychological symptoms were assessed by questionnaire rather than clinical diagnostics. However, SDQ is a reliable scale which has been validated and widely used in China. [21] Still, the presence of abnormal defined by SDQ scores is a good predictor of diagnosed mental health disorders. [31] Third, it is possible that our results might be affected by unmeasured factors such as parents’ mental health disorders, although we had controlled for several confounders. Parents’ mental health disorders might also play a role in the development of mental health disorders in offspring during childhood. [32] Fourth, the nature of these data are cross-sectional. Although causal inference could not be established, the direction between SHS exposure and psychological symptoms is robust. Fifth, missing data are inevitable in the large population-based study even though the percentage of missing is not very high. However, multiple imputation was used for missing exposures and confounders in the model to increase statistical power. [24] Sixth, we do not have information on children’s lifestyle (i.e., smoking or alcohol use). Children with higher SHS exposure are more likely to initiate smoke or drink. Inability to control for these confounders might overestimate our associations. However, in China, the prevalence of smoking among primary school students are very lower (less than 0.2%). [33] Lastly, the association between SHS exposure and worse psychological outcomes among children could be explained in part by prenatal exposure to tobacco. [34] Unfortunately, the lack of prenatal tobacco exposure measurement prevented us from examining this impact. For maternal SHS from home sources, due to the fact that smoking behavior is often difficult to change, children who are exposed to SHS at home are likely to have been exposed to SHS in utero. In these cases, due to the high correlation between in utero and postnatal exposure to SHS, [34] the observed association remains true. In cases where maternal SHS exposure was from the workplace, fetal exposure may have occurred but the child may not be exposed to SHS. Taking all these scenarios into account, the association between child SHS exposure and psychological symptoms in our study is likely to be underestimate. Nonetheless, our study is unable to distinguish between fetal and child exposure to SHS. Pre-and postnatal exposure to tobacco are highly correlated and difficult to differentiate. [34]

Several potential mechanisms may explain our findings. First, nicotine, the main psychoactive component in tobacco, can exert neurotoxic effects on developing brain areas involved in emotional processing. [35] Nicotine exposure mediates the acetylcholine receptor through activating and desensitizing neuronal nicotinic acetylcholine receptors (nAChRs). [35] During primary school years, children’s brain are still undergoing dramatic changes which are vulnerable to the harmful effect of nicotine exposure. [36] Second, catecholaminergic neurotransmitter systems may also provide a link between SHS exposure and psychological symptoms. The activation of nicotinic acetylcholine receptors can affect the catecholaminergic neurotransmitter system and the level of catecholamine dopamine and noradrenaline in the prefrontal cortex and independently regulate attention and behavioral symptoms. [37] Third, the observed estimation between SHS exposure and psychological symptoms could be explained by the passive gene-environmental correlation. [38] For example, mothers who pass down the genetic variants for psychological symptoms to their children might also have an increased risk of being exposed to SHS. SHS exposure could more likely to be considered as genetic risk that parents transmit to children rather than a risk factor for a child’s psychological symptoms. Fourth, we cannot rule out the possibility that the observed associations could be due to residual confounding by SES, other uncontrolled confounding such as unhealthy lifestyles, or fewer social and psychological resources available or the biological effect of SHS. [39] Furthermore, in-utero tobacco exposure might also play a role in the association. [40] Several studies have found that children exposed to tobacco during the prenatal period have increased risk of developing more behavioral and emotional problems. [41–43]

## Conclusion

In summary, our study has confirmed previous studies showing SHS exposure is associated with more psychological symptoms among children. Even though we could not rule out the possibility that prenatal exposure to SHS might also contribute to the observed associations, our result has important public health implications. Children’s psychological symptoms has constituted a global epidemic. Identifying modifiable risk factors will facilitate effective prevention strategies. Smoke free policy is the most effective way to reduce SHS exposure at public and private places. Efforts to restrict SHS exposure in public places or homes where children are present could provide a perspective to alleviate the heavy burden of diseases attributable to cigarette smoking. Our findings could be used to inform future SHS control policies and reinforce the need for public education campaigns. These campaigns could be implemented to raise the public awareness of the harmful psychological effect of SHS exposure.

## Additional file

Additional file 1: **Table S1.** Adjusted associations of secondhand smoke exposure with psychological symptoms in the SCHEDULE study in China. (DOCX 16 kb)

## Data Availability

The datasets used and/or analyzed during the current study are available from the corresponding author on reasonable request.

## Abbreviations

CI: Confidence intervals
SCHEDULE: Shanghai Children’s Health, Education and Lifestyle Evaluation
SD: Standard deviations
SDQ: Strengths and Difficulties Questionnaire
SES: Socio-economic status
SHS: Secondhand smoke
WHO: World Health Organization
