# Supplementary material for: The association between exposure to secondhand smoke and psychological symptoms among Chinese children
Source: BMC Public Health. 2019 Jul 10;19:923. doi: 10.1186/s12889-019-7006-8 (PMC6617699; doi:10.1186/s12889-019-7006-8)
Supplement: Supplementary file 1 — Table S1. Adjusted associations of secondhand smoke exposure with psychological symptoms in the SCHEDULE study in China. (DOCX 16 kb) [file 12889_2019_7006_MOESM1_ESM.docx]

Additional file 1: Table S1 Adjusted associations of secondhand smoke exposure with mental disorder in the SCHEDULE study in China

|  |  |  | Secondhand smoke exposure | | |
| --- | --- | --- | --- | --- | --- |
|  |  | None | <1 hour/daily | 1-2 hours/daily | ≥3 hours/daily |
|  |  | Reference | OR (95% CI) | OR (95% CI) | OR (95% CI) |
| Model 1 |  |  |  |  |  |
| Total mental disorders | | 1 | 1.69 (1.38 to 2.06) | 2.37 (1.83 to 3.06) | 2.83 (2.12 to 3.79) |
| Emotional symptoms | | 1 | 1.32 (1.06 to 1.64) | 1.70 (1.27 to 2.27) | 1.87 (1.33 to 2.61) |
| Conduct problems | | 1 | 1.34 (1.08 to 1.65) | 2.08 (1.57 to 2.74) | 2.18 (1.59 to 3.00) |
| Hyperactivity-inattention | | 1 | 1.41 (1.22 to 1.63) | 1.66 (1.36 to 2.04) | 2.29 (1.81 to 2.90) |
| Peer relationship problems | | 1 | 1.30 (1.15 to 1.47) | 1.70 (1.43 to 2.03) | 1.32 (1.06 to 1.65) |
| Prosocial behaviors | | 1 | 1.10 (0.89 to 1.36) | 1.32 (0.98 to 1.77) | 1.32 (0.92 to 1.89) |
| Model 2 |  |  |  |  |  |
| Total Mental disorders | | 1 | 1.61 (1.22 to 2.12) | 2.30 (1.62 to 3.27) | 2.57 (1.70 to 3.89) |
| Emotional symptoms | | 1 | 1.45 (1.06 to 1.99) | 1.89 (1.26 to 2.82) | 2.10 (1.31 to 3.36) |
| Conduct problems | | 1 | 1.14 (0.85 to 1.51) | 1.48 (1.01 to 2.17) | 2.21 (1.43 to 3.41) |
| Hyperactivity-inattention | | 1 | 1.41 (1.16 to 1.71) | 1.47 (1.12 to 1.94) | 2.42 (1.76 to 3.34) |
| Peer relationship problems | | 1 | 1.25 (1.06 to 1.49) | 1.62 (1.27 to 2.08) | 1.37 (1.01 to 1.86) |
| Prosocial behaviors | | 1 | 1.25 (0.93 to 1.69) | 1.41 (0.95 to 2.09) | 1.85 (1.14 to 3.00) |

Model 1 adjusted for sex, age at measurement;

Model 2 additionally adjusted for parents’ education, household income, mode of birth, place of birth and BMI z-score (relative to the 2007 World Health Organization growth reference)
